# Mr. Watt, in Reply to Mr. Sheldrake

**Published:** 1800-12

**Authors:** R. Watt

**Affiliations:** Paisley


### *To the Editors of the Medical and Phyfical Journal.*

Gentlemen,

In a late Number of your valuable Journal, I obferve that a Mr. Sheldrake has given what he denominates an anfwer to my Communication on diftorted limbs. Regard for the interefts of truth undoubtedly induced you to publifh his anfwer; and refpect to juftice will induce you, I hope, to admit my reply.

The general common-place obfervations in the firft part of the anfwer are, in my opinion, an odd mixture of affected candor and of intentional feverity. As they, however, affect not the merits of my communication, but, like many introductions, may with fafety be attached to any other publication, I fhall proceed to confider that part of the anfwer which calls the originality of the invention in queftion. For this, three reafons, befide his own claim, which is afterwards *modeftly* advanced, are propofed.

1. Mr. S. fays, he has fome *reafons* to conclude that the invention is not very diffimilar from the *Edinburgh boots*, which, by the bye, he does not pretend to have feen, but only received, by fecond hand, a defcription of them which came from a Dr. Deafe, with whom he had no communication on the fubject. The want of *candor* in this firft reafon is certainly inconfiftent with that *difplay* of it fo frequently made by Mr. S. And if he who has made the fubject of diftorted limbs fo much his ftudy, has not met with thefe boots, it cannot be furprifing that they never came in the way of a practitioner in the *country*?—

What

What thefe *well-known* Edinburgh boots were I have not been able to learn, nor can I form any idea of them from the account Mr. S. has given, further than that they *prefs* and *comprefs* the legs; and from this *candid*, but certainly *fuperficial* mode of reafoning, he concludes that it is impoffible they can be very *diffimilar* to the one in queftion.

2. The circumftance of Dr. Aitkin's inftrument refembling mine in *part*, is adduced as the fecond reafon; and I may be allowed to obviate this objection by obferving that the Doctor's inftrument was not to my purpofe, and has not anfwered the purpofe of furgeons in general. In the firft of the two cafes I have communicated to the public, before the application of my machine, and before I faw the cafe, Dr. Aitkin's invention, as well as many others, had been repeatedly tried, but without fuccefs. With regard to the fimilarity between the two contrivances I fhall fay nothing; the Doctor's Effays on Fractures, &c. are well known, and every one has it in his power to compare and judge accordingly.

3. It may appear a wafte of time to refute his third reafon, fince Mr. S. admits that the invention of Mr. Holmes, the fhoe-maker, only *imperfectly* cured fome very *flight cafes*, but was never able *to cure any one important cafe of diftortion.* Why then, fince his invention is of fo little confequence, is it brought into view? I fhould certainly have fufpected, had Mr. S. been writing another *Practical Effay* on diftorted limbs, that he had foifted in the *fhoe-maker* with little other motive than merely to *swell* the fize of his volume.

Thus it appears that the *reafons* Mr. S. propofes againft the originality of the invention are nothing more than *bare affertions.* The firft is founded upon *report.* The fecond, even according to his own account, which, by the bye, is carried to the extreme, only *refembles it*; nay ftill lefs, only *refembles it in part.* And the third, where the *fhoe-maker* is brought into view, is ftill more trifling; for, granting that there is fuch a man as Mr. Holmes, the fhoe-maker, and that he has invented fomething for curing diftorted limbs, it appears, to ufe the words of Mr. S. that his invention was totally ufelefs, having never cured any one important cafe of diftortion. What their *long* experience proves I cannot tell; but I beg leave to fay, that my *fhort* experience has witneffed the cure of *more than one* important cafe of diftortion. Indeed, with regard to the labours of Mr. Holmes, the *fhoe-maker*, and with him I may affociate Mr. Sheldrake, the *trufs-maker*, it would be *uncandid*, nay *unjuft*, to fay any thing, having never feen, much lefs tried, the invention either of the one or the other.

Having

Having faid *thus much* of the *originality* of the invention, Mr. S. introduces his remarks on its *efficacy* with a few more common-place obfervations, which, as they appear to be little to the point, I fhall pafs over, and proceed to confider his *affertions* with regard to its *inutility*.—He cannot conceive how any man, and particularly one at fuch a *diftance* from the metropolis, could have the *temerity* and *confidence* to thruft down people's throats fuch *crude* and indigeftible notions; notions which have no other foundation in practice than two cafes, and which Mr. S. in his ufual *hypothetical* ftile, affures his readers were only *very flight ones.*—If it is proceeding upon flight and fuperficial ground, to venture to recommend a method of cure which has been completely fuccefsful in two cafes, it is certainly ftill more fo to condemn that method, without giving it a trial at all. This is certainly not like the actions of a man of *candor*, who proceeds by *reiterated experiments*, and whofe opinions and affertions are founded alone on the evidence of facts. Yet fuch is the conduct of this hero, who has fo humanely and *difinterefledly* volunteered his fervices to protect the healing art from fuch *crudities* and grofs innovations.

There is one thing in Mr. S's anfwer, which the moft fuperficial reader muft have obferved with aftonifhment, that is, the wonderful verfatility of his genius in accommodating itfelf, with fo much facility, to all the endlefs varieties of this clafs of difeafes. He affures us, that of more than an hundred cafes which he has cured perfectly, no two were alike, or could have been cured without adopting the mode of treatment peculiar to each. Nay, what is ftill more aftonifhing, there is not only this endlefs variety in the cafes themfelves, but *there is, in every cafe, one condition of the bones that will require peculiar treatment, there is another condition of the ligaments, and a third of the mufcles.* To believe the firft of thefe affertions, that no two cafes are *exactly* alike, requires no uncommon ftretch of credulity; and the laft, although it may excite the fmile of the anatomift, certainly adds little to our knowledge of the *facts* of the difeafe.

Mr. S. has faid much about *crude* ideas, and *crude* notions, of the *facts* of this difeafe. I fhould be glad to know in what particular he has rendered our ideas of the *facts* of the difeafe more precife, fimple, or intelligible than they were; and where he has fubftituted better terms than thofe of VARUS and VALGUS, which have been fo univerfally ufed. I am of the fame opinion with him, that terms fhould be as appropriate as poffible, and that *varus* and *valgus*, if not fufficiently defcriptive, be rendered explicit by definition, or give way to terms better adapted adapted to determine the nature of the difeafe. But I cannot furely incur any blame for employing the old terms, which Mr. S. with all the extenfive information to which he lays claim, has not ventured to expunge. If my ideas of the facts of the difeafe *feem* to be *crude*, my practice has been *really* fuccefsful; and Mr. S's *mite*, I am affured, has not added much to the intelligence of any of my medical friends who have read his anfwer. Indeed, the only thing that can be gathered from the whole of Mr. S's anfwer, is a conftant wifh to magnify the difficulties of applying the machine, and effecting a cure, and might, perhaps, have been expreffed in a few words, by fimply telling us, *that none could do any thing to it but himfelf.*

*Thefe affertions, the refult of Mr. Sheldrake's many years practice, fet this clafs of difeafes in a light they had never been placed in by any perfon whatever, and he has thus difcovered and perfected a method of cure much more effectual than any that had previoufly been known!* To fay nothing of the *modefty* of this claim, I fhall only obferve, that if there be one condition of the bones, another of the ligaments, and a third of the mufcles, that require treatment peculiarly adapted to each; and if no two cafes in more than an hundred be alike, or could have been cured by the fame means, the public can derive little or no advantage from the publicity of his patent, or the application of his machine, which, in this cafe, would require a verfatility of genius to adapt it, equal, if not fuperior, to that of its *Original Inventor.*

In oppofition to this eccentric mode of reafoning, I can affure Mr. S. that I have now cured two cafes completely, and at this moment I have proceeded a confiderable length with fome more; that all of them were, in fome refpects different; but all are likely to be cured by the fame means. And if I may now believe the teftimony of my fenfes, as well as the opinion of fome eminent medical gentlemen, I may fay, and with more confidence than ever, "that there is no cafe of diftorted limbs, however formidable they may appear, *if taken in time*, but may be cured by the fame means." I fay, *if taken in time*, for my experience does not authorife me to fay, that one above a year and a half old can be cured of the difeafe.

In a fubfequent part of the anfwer, I was fomewhat furprifed to find, a refemblance mentioned between my invention and that of Mr. S. He is very careful to expofe the inefficacy of mine; and this affords a clue by which we can fee through the ambiguity of his expreffions, and arrive at the motive of his attack. But prudence fhould have dictated another line of conduct, fince, by cenfuring one whom he confiders a rival, he expofes himfelf to fimilar condemnation. What he he adduces againſt the inefficacy of my invention, if the reſemblance be ſtriking, muſt be equally applicable to his. I am far from thinking that either are ſo ineffectual as Mr. S. would inſinuate: I have informed the public of the effect produced by the inſtrument which I contrived; and as I have not had an opportunity of applying Mr. Sheldrake's, I cannot in juſtice give any opinion upon its merits. I did not expect that a man of *candor* would have commenced an attack upon any invention till he had made trial of its merits, and by the *ſure teſt* of EXPERIMENT been enabled to decide with *accuracy*. The *crudeneſs* of this manner of cenſuring, is obvious without a comment; and though, from my ſhort ſtanding in the profeſſion, I cannot lay claim to Mr. Sheldrake's OMNISCIENCE in caſes of diſtortion, I affirm, that his anſwer has done very little to render our knowledge of the *facts* of the diſeaſe more complete than they were before.

I may obſerve too, that if my inſtrument nearly reſembles Mr. Sheldrake's, it is equally efficacious; if more imperfect, it need not have given him alarm; and if an improvement on his conſtruction, as a friend to humanity, he ſhould have rejoiced in what was likely to benefit mankind. Had Mr. S. been convinced in his own mind that he could ſubſtantiate his charge of my invention being the ſame, or ſimilar to his, he had no occaſion to employ this method of procuring redreſs. No, have recourſe to his *patent*, which, he ſays, he obtained to ſecure the benefit of his diſcovery to *himſelf*. Let him have recourſe to the laws of his nation, and ſeek redreſs in its proper channel, and not with his native effrontery *criminate* thoſe by BARE ASSERTIONS whom he cannot *condemn* by SOUND ARGUMENT.

The *candid manner* in which my Communication has been treated, lays me under under ſtrong obligations to ſpecify the particulars moſt deſerving of my gratitude. I conſidered it as neceſſary to mention the minuteſt circumſtances attending the cure; and when humanity and caution dictated the preſcription of a mild opiate, I did not expect that for this I would have received animadverſions from any perſon who had the moſt diſtant pretenſions to medical ſkill. If Mr. S. has cured more than a hundred important caſes of diſtortion without adminiſtering any medicine to alleviate the pain neceſſarily attendant on the progreſs of the cure, he has been fortunate indeed, or is poſſeſſed of feelings not very tenderly alive to infantine ſuffering. In the firſt caſe I found it neceſſary; in the ſecond, although the caſe was conſiderably worſe, I did not: In one of them I have under my care at preſent, I uſe it occaſionally; in the reſt, I find it abſolutely unneceſſary.

An

An error, either typographical or in the MS. tranfmitted, has met with cenfure. The flighteft reflection might have difcovered, that the fecond NOT in line 8, page 94, was inadvertently inferted; and on this account it was certainly below any man, much more a man of *candor*, to have taken the advantage of it.

I take leave of this fubject, requefting that my readers will pardon any appearance of *egotism* that may appear in this reply, fince it is not eafy to talk of onefelf without giving offence. I am,

GENTLEMEN,

Your much obliged fervant,

*Paifley, Oct.* 21, 1800. R. WATT.

*** In a private letter from Mr. Watt, he fays, "In confequence of my former communication, I have had many applications of late from different parts of the kingdom, and have at prefent under my care a great many cafes of diftortion. I have, fince my laft communication, invented another machine, which I apply when there is only one foot diftorted, and when the patient is fo old as to be able to walk. This machine retains the limb in a proper pofition, while at the fame time the patient can walk about with tolerable eafe. By making a little alteration, I have alfo applied it with great advantage to debility of the knee and ankle joint, and for removing ftiffneffes of the knee-joint, occafioned by contraction of the mufcles and tendons on the pofterior fide of the limb: An account of this will probably form the fubject of a future communication. I believe, in a former letter I informed you, that I had invented a machine for curing diftortions of the fpine, fhoulders, and neck; upwards of twenty of which have now been made, and applied with great advantage.

"In a letter a few days ago, which I had from Mr. Moyes, of Edinburgh, he informs me, that he has lately had orders from York for two of the inftruments for Lithotomy, (publifhed in No. XIII. of your Journal) one to Aberdeen, and two to two medical gentlemen in Edinburgh. I have myfelf, within thefe three months, had orders for one to Manchefter, one to Liverpool, one to Glafgow, and one to Philadelphia. From which demands, I flatter myfelf, that fome time or other it may come into general ufe.

